# Evaluation of endocervical curettage (ECC) in colposcopy for detecting cervical intraepithelial lesions

**DOI:** 10.1007/s00404-024-07721-w

**Published:** 2024-10-29

**Authors:** Annika S. Behrens, Anna K. Dietl, Werner Adler, Carol Geppert, Arndt Hartmann, Antje Knöll, Matthias W. Beckmann, Grit Mehlhorn, Martin C. Koch, Carla E. Schulmeyer, A. Seibold, Paul Gass, Frederik A. Stuebs

**Affiliations:** 1 Department of Gynecology and Obstetrics, Comprehensive Cancer Center Erlangen–European Metropolitan Area of Nuremberg (CCC ER-EMN), Friedrich-Alexander-Universität Erlangen–Nürnberg, Erlangen University Hospital, Universitaetsstrasse 21–23, 91054 Erlangen, Germany; 2 Department of Medical Informatics, Biometry and Epidemiology, Friedrich Alexander University of Erlangen–Nuremberg, Waldstrasse 6, 91054 Erlangen, Germany; 3 Institute for Pathology, Comprehensive Cancer Center Erlangen–European Metropolitan Area of Nuremberg (CCC ER-EMN), Friedrich-Alexander-Universität Erlangen–Nürnberg, Erlangen University Hospital, Krankenhausstrasse 8–10, 91054 Erlangen, Germany; 4 Institute of Clinical and Molecular Virology, Friedrich-Alexander-Universität Erlangen–Nürnberg, Erlangen University Hospital, Schlossgarten 4, 91054 Erlangen, Germany; 5 Gynecology Consultancy Practice, German Cancer Society (DKG) and Working Group on Cervical Pathology and Colposcopy (AGCPC) Certified Gynecological Dysplasia Consultancy Practice, Frauenarztpraxis Erlangen, Neustädter Kirchenplatz 1a, 91054 Erlangen, Germany; 6 Department of Gynecology and Obstetrics, ANregiomed Ansbach Hospital, Escherichstrasse 1, 91522 Ansbach, Germany

**Keywords:** Cervical intraepithelial neoplasia, Endocervical curettage, Colposcopy, Cervical cancer, Cervical cancer screening assessment, Cytology

## Abstract

**Purpose:**

Diagnostic challenges in colposcopy arise especially in women aged 50 or older, with postmenopausal status and transformation zone type 3 (TZ3). Endocervical curettage (ECC) is a valuable tool for diagnosing intracervical lesions. The aim of this retrospective analysis was to evaluate the use of ECC in colposcopy for detecting cervical intraepithelial lesions.

**Methods:**

A retrospective study was carried out of colposcopies performed in the certified Dysplasia Unit at Erlangen University Hospital between July 2016 and June 2023. Pap and human papillomavirus (HPV) results were correlated with the histologic findings via ECC, obtained during examinations or surgery. The primary outcome was the rate of accuracy between the colposcopic and histologic findings with regard to cytology, age of patients, and type of transformation zone (TZ).

**Results:**

A total of 429 colposcopies in 413 women with histologic samples obtained via ECC were included in the final analysis. In all, 355 women had TZ3. Among patients with TZ3, evidence of high-grade lesions and invasive carcinoma was also found in women with normal or low-grade abnormal cytology. For patients with normal colposcopic findings, cervical intraepithelial neoplasia (CIN) 2 and CIN 3/adenocarcinoma in situ (AIS) were found in 56 patients (16%), and invasive carcinoma was found in four patients (0.1%).

**Conclusion:**

This analysis suggests that ECC is a valuable tool in the diagnosis of cervical intraepithelial neoplasia, especially for patients who present with a normal colposcopy of the cervix and vagina but have either recurrent abnormal cytologic findings or high-grade abnormal cytology indicating CIN 2 + .

**Supplementary Information:**

The online version contains supplementary material available at 10.1007/s00404-024-07721-w.

## What does this study add to the clinical work

**Table:** 

The present study provides valuable information regarding the use of endocervical curettage (ECC) as a valuable tool in the diagnosis of endocervical intraepithelial neoplasia. The data indicate that performing ECC should be considered in patients with persistent hrHPV positivity and normal cytology as well as in patients with TZ3.	

## Introduction

Cervical cancer is among the most common cancer entities among women worldwide [1–3]. Over recent decades, the incidence of cervical cancer has significantly declined in developed countries. This is mainly attributed to the effect of nationwide screening programs [4, 5]. Precancerous lesions such as high-grade squamous lesions (HSIL) are typically caused by persistent infection with human papillomavirus (HPV) and can progress to invasive cervical cancer [6–8]. Detecting HSIL thus plays a crucial role in the prevention of cervical cancer. Women participating in nationwide screening programs are regularly examined by their gynecologist, depending on their age. Cytology and HPV test results are obtained during these examinations. Cytology is a highly accessible, feasible, and cost-effective screening tool for HSIL and invasive cervical cancer [9, 10].

In Germany, women with abnormal cytology findings are referred to certified dysplasia units [8, 11], where colposcopy is performed for further evaluation. Colposcopy is a cost-effective assessment tool for accurate diagnosis of HSIL in women with abnormal cytology and/or positive high-risk human papillomavirus (hrHPV) findings. It allows an experienced examiner to identify and localize lesions, estimate the lesion’s severity, and obtain colposcopically-directed biopsies [12].

However, colposcopy has severe limitations for evaluating the endocervical canal. The majority of cervical intraepithelial neoplasia (CIN) lesions are located in the cervical transformation zone (TZ). In 2011, the International Federation for Cervical Pathology and Colposcopy (IFCPC) introduced a classification comprising three different types of TZs, categorizing the squamocolumnar junction into “visible,” “partially visible,” or “not visible.” A type 1 transformation zone (TZ1) is located entirely on the ectocervix; a type 2 zone (TZ2) has endocervical parts, but is visible as a whole when technical aids are used to widen the cervical canal; and a type 3 transformation zone (TZ3) has partial or complete endocervical involvement and is not visible in its entirety. In cases of TZ3, the whole extent of major lesions may not be visible. A statement about the TZ is therefore considered obligatory in a colposcopic assessment [13, 14].

A recent meta-analysis drew attention to diagnostic challenges especially in women aged 50 or older, with postmenopausal status and TZ3 [15]. In a substantial number of cases, inspection of the endocervix and application of 5% acetic acid is not possible. Particularly in patients with TZ3, colposcopy cannot be carried out adequately and it is often impossible to obtain representative histologic samples using a directed biopsy [16]. In these patients, histologic evaluation using endocervical curettage (ECC) may be necessary, taking the patients’ individual risk pattern into account. ECC is a low-risk, low-morbidity procedure that is widely available and also time- and cost-effective. According to the existing German guidelines of the Federal Joint Committee (G-BA), ECC should be performed, if medically indicated, for patients with a TZ3 [17]. According to the 2017 Colposcopy Standards Consensus Guidelines of the American Society for Colposcopy and Cervical Pathology (ASCCP) and a recent update article, ECC is recommended in several patient scenarios, such as when CIN 2 is detected, to rule out intracervical CIN 3 + , and to determine whether a conservative or a surgical approach may be feasible; when there is known hrHPV type 16 or 18 infection; abnormal cytology in patients with a history of HSIL or carcinoma; suspicion of CIN 3/adenocarcinoma in situ (AIS) or invasive cancer; the presence of TZ3 at colposcopy; and others [18, 19]. These guidelines state that, although ECC is obsolete in pregnant patients, it is acceptable for all nonpregnant patients undergoing colposcopy [19]. Data on routinely performed ECC is inconclusive. Studies suggest that the rate of additional diagnosis of CIN 2 + by means of ECC is between 1 and 13% [20, 21]. The benefits of ECC may be greater in older women [21]. Other studies report that changes in treatment plans on the basis of additional ECC are only made in a small group of patients [18, 22].

The aim of this retrospective analysis was to evaluate the use of ECC in colposcopy for detecting endocervical cervical intraepithelial lesions.

## Materials and methods

**Fig. 1:**
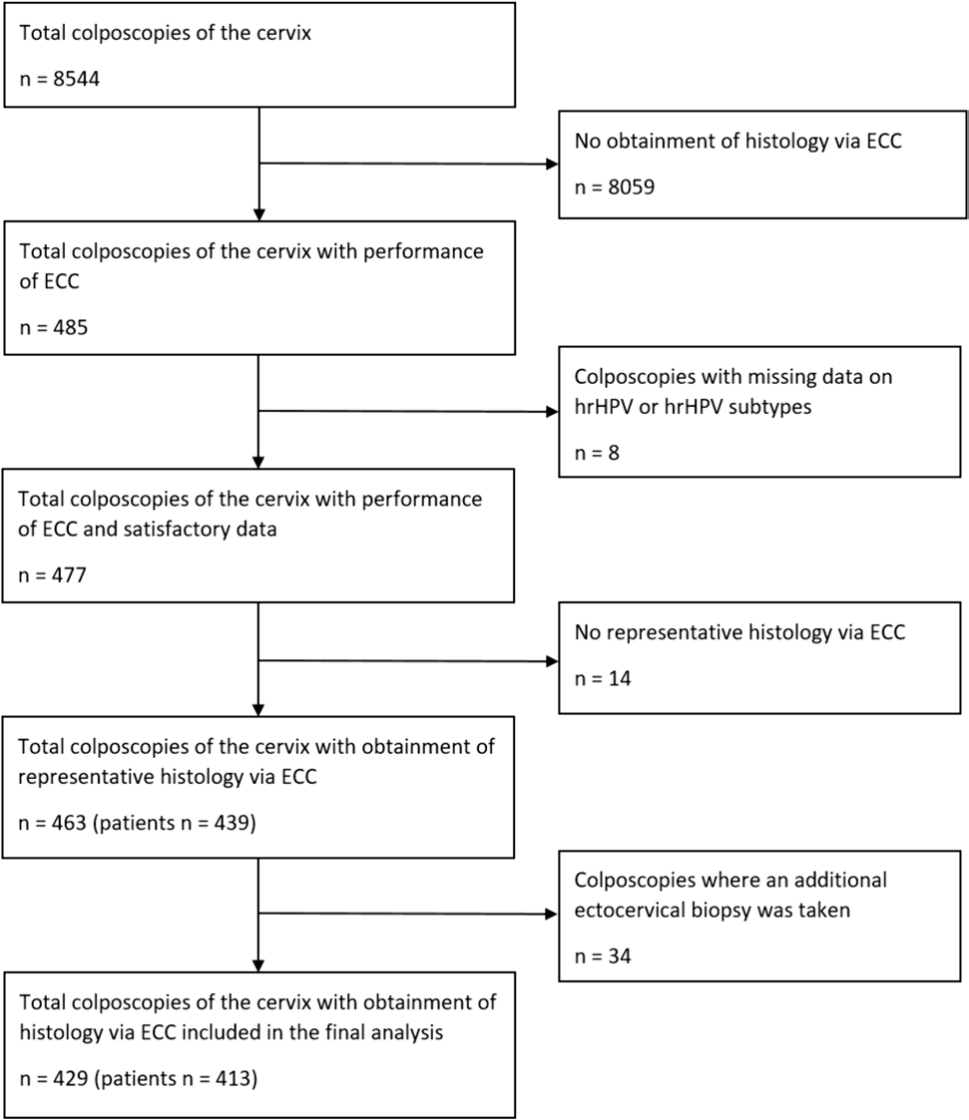
Flowchart

We retrospectively assessed colposcopies of the cervix in which histology samples had been obtained via ECC. All patients who underwent ECC were identified. A total of 485 patients seen at the nationally certified dysplasia unit at Erlangen University Hospital between July 2016 and June 2023 were eligible for further assessment. Cases were excluded in which representative histology was not obtained during the ECC, as well as cases in which no further information about hrHPV status was available or an additional ectocervical biopsy was taken. We omitted cases in which an additional ectocervical biopsy was taken. This was done to minimize the risk of bias and to enable a clear assignment of the histology to ECC. In total, 429 colposcopies in 413 women were assessed in the final analysis (Fig. 1). In cases in which the histologic findings differed between the ECC and surgery, the most severe histology was included in the statistical analysis.

At the nationally certified dysplasia unit at Erlangen University Hospital, colposcopies are performed in standardized conditions using a Zeiss KSK 150 FC colposcope. General assessments are performed in accordance with the 2011 International Federation for Cervical Pathology and Colposcopy (IFCPC) colposcopic terminology for the cervix [14, 23, 24]. The standard of care procedures in our dysplasia unit include conventional cytology (Pap smear of the cervix), testing for HPV—hybrid capture test 2 (2015–2018); Abbott RealTime high-risk HPV assay on an Abbott m2000sp (2019–2020) or Roche, cobas®4800, HPV Test, Multiplex-RT-PCR (since 2020)—and application of 5% acetic acid to the cervix a well as Lugol’s iodine in some cases, these procedures have been reported elsewhere [25–28].

Colposcopic findings are classified in accordance with the IFCPC into “normal” or “abnormal”—subdivided into “minor” or “major” and “suspicious for invasion/cancer” [14, 23, 24]. In patients with major findings or lesions suspicious for invasion, a colposcopy-directed biopsy has to be taken from the most conspicuous part of the lesion. In some patients with multifocal lesions, more than one biopsy is necessary [8]. ECC has since October 2021 been performed on the basis of the patients’ individual risk pattern using a curette (Kevorkian 310–727-001; Eisenhut Instrumente GmbH, Frittlingen, Germany). Before that, another curette (Recamier Uterine Curette; Aesculap AG, Tuttlingen, Germany) was used in our unit. The indication for ECC in the dysplasia unit includes patients who present with a normal colposcopy of the cervix and vagina, but have either recurrent abnormal cytologic findings or high-grade abnormal cytology indicating CIN 2 + . Other cases comprise patients with persistent hrHPV positivity and normal colposcopic findings, or lesions with suspicion of invasion.

All surgical treatments were performed by experienced and highly qualified staff at Erlangen University Hospital in standardized conditions [4].

All patient data, including colposcopic findings, Pap smear and HPV test results, histologic outcomes, numbers and method of biopsies performed, type of TZ, and epidemiologic data were recorded prospectively in a database for further research. For patients in whom subsequent surgery of the diagnosed lesions was performed, the histologic results at surgery were also collected and analyzed. In cases in which histologic findings differing between the ECC and surgery were detected, the most severe histology was included in the statistical analysis.

The accuracy rate is the percentage of patients with the same colposcopic findings and more severe histology: normal, miscellaneous, and unspecific colposcopic findings are considered equivalent to benign histology, minor findings to CIN 1/low-grade intraepithelial lesion (LSIL), and major findings to CIN 2/CIN 3/AIS/HSIL, while suspected invasion correlates with invasive cancer.

### Statistical analysis

For statistical analysis, the different Pap grades were subdivided into four different groups (testing for HPV was carried out on all Pap smears) (Table S1). The histologic findings were subdivided into two groups: LSIL − (benign and CIN 1/LSIL) vs. HSIL + (CIN 2/HSIL, CIN 3/HSIL; AIS/HSIL; carcinoma) [8].To evaluate the relationship between the histology and Pap findings, we modeled a logistic regression with dichotomous histology (LSIL −  vs. HSIL +) as dependent variable and Pap as independent variable. To examine the influence of hrHPV testing, HPV was added as an additional independent variable in a second logistic regression model. As there were 429 observations in 413 patients, generalized estimating equations (GEE) were used to correctly estimate the *P* values in these models. Odds ratios, 95% confidence intervals, and *P* values are reported. The level of significance is set to 0.05. With these models, the probabilities for HSIL + were calculated and receiver operating characteristic (ROC) analysis was performed. The areas under the ROC curve (AUC) were calculated and compared using the DeLong test. A somewhat more complex bootstrap analysis was performed to rule out bias due to the existing dependency between some of the observations. This led to comparable results (Fig. S1 and S2). All statistical analyses were carried out using the R statistical package, version 4.3.1 [29].

## Results

Four hundred and twenty-nine colposcopies in 413 women were included in the final analysis (Fig. 1).

Table 1 shows the overall results for Pap in correlation with histology in ECC or surgery for all 429 cases in which ECC was performed. Information on hrHPV status for these results is available in the supplementary materials (Tables S2-5). In all, 249 cases tested positive for hrHPV, while 180 cases tested negative.

**Table 1:** Papanicolaou smear (Pap) in correlation with the most severe histology in endocervical curettage or surgery

Total Pap smears (*n* = 429, 413 patients)	Bethesda system	Benign (*n* = 269)	CIN 1/ LSIL (*n* = 75)	CIN 2/ HSIL (*n* = 23)	CIN 3/ AIS/ HSIL (*n* = 54)	Carcinoma (*n* = 8)
I (*n* = 84)	NILM	68 (81%)	11 (13%)	2 (2%)	3 (4%)	0
II-a (*n* = 59)	NILM	52 (88%)	6 (10%)	1 (2%)	0	0
II-g (*n* = 4)	AGC endocervical NOS	3 (75%)	1 (25%)	0	0	0
II-p (*n* = 108)	ASC-US	80 (74%)	18 (17%)	7 (6%)	2 (2%)	1 (1%)
IIID1 (*n* = 86)	LSIL	48 (32%)	29 (34%)	5 (6%)	4 (5%)	0
IIID2 (*n* = 33)	HSIL	6 (18%)	4 (12%)	6 (18%)	16 (48%)	1 (3%)
III-g (*n* = 2)	AGC endocervical, favoring neoplastic	2 (100%)	0	0	0	0
III-p (*n* = 25)	ASC-H	4 (16%)	6 (24%)	1 (4%)	12 (48%)	2 (8%)
IVa-p (*n* = 19)	HSIL	5 (26%)	0	1 (5%)	13 (68%)	0
IVa-g (*n* = 3)	AIS	1 (33%)	0	0	1 (33%)	1 (33%)
IVb-p (*n* = 2)	HSIL with features suspicious for invasion	0	0	0	2 (100%)	0
IVb-g (*n* = 1)	AIS with features suspicious for invasion	0	0	0	0	1 (100%)
V-p (*n* = 2)	Squamous cell carcinoma	0	0	0	1 (50%)	1 (50%)
V-x (*n* = 1)	Other malignant neoplasms	0	0	0	0	1 (100%)

*AGC* atypical glandular cells, *AIS* adenocarcinoma in situ, *ASC-H* atypical squamous cells high-grade squamous intraepithelial lesion cannot be excluded, *ASC-US* atypical squamous cells of undetermined significance, *CIN* cervical intraepithelial neoplasia, *HSIL* high-grade squamous intraepithelial lesion, *LSIL* low-grade squamous intraepithelial lesion, *NILM* negative for intraepithelial lesion or malignancy, *NOS* not otherwise specified

Twenty-five women had a TZ1 at the time of colposcopy, while 49 women had a TZ2 and 355 women had a TZ3. Data on Pap and HPV findings in combination with histology at ECC or surgery for patients with TZ1 and TZ2, as well as for patients with TZ3 are shown in Tables 2 and 3. A detailed breakdown of patients with TZ1 and TZ2 can be found in the supplementary materials (Tables S3 and S4).

**Table 2:** Papanicolaou smear (Pap) in correlation with the most severe histologic finding in endocervical curettage or surgery for patients with a type 1 transformation zone (TZ1) or type 2 transformation zone (TZ2)

Total Pap smears (*n* = 74)	Bethesda system	Benign (*n* = 51)	CIN 1/ LSIL (*n* = 7)	CIN 2/ HSIL (*n* = 6)	CIN 3/ AIS/ HSIL (*n* = 8)	Carcinoma (*n* = 2)
I (*n* = 13)	NILM	13 (100%)	0	0	0	0
II-a (*n* = 12)	NILM	9 (75%)	2 (17%)	1 (8%)	0	0
II-g (*n* = 2)	AGC endocervical NOS	2 (100%)	0	0	0	0
II-p (*n* = 16)	ASC-US	13 (81%)	1 (6%)	2 (13%)	0	0
IIID1 (*n* = 16)	LSIL	10 (63%)	3 (18%)	2 (13%)	1 (6%)	0
IIID2 (*n* = 7)	HSIL	2 (29%)	0	1 (14%)	4 (57%)	0
III-p (*n* = 1)	ASC-H	0	1 (100%)	0	0	0
IVa-p (*n* = 4)	HSIL	2 (50%)	0	0	2 (50%)	0
V-p (*n* = 2)	Squamous cell carcinoma	0	0	0	1 (50%)	1 (50%)
V-x (*n* = 1)	Other malignant neoplasms	0	0	0	0	1 (100%)

*AGC* atypical glandular cells, *AIS* adenocarcinoma in situ, *ASC-H* atypical squamous cells high-grade squamous intraepithelial lesion cannot be excluded, *ASC-US* atypical squamous cells of undetermined significance, *CIN* cervical intraepithelial neoplasia, *HSIL* high-grade squamous intraepithelial lesion, *LSIL* low-grade squamous intraepithelial lesion, *NILM* negative for intraepithelial lesion or malignancy, *NOS* not otherwise specified

**Table 3:** Papanicolaou smear (Pap) in correlation with the most severe histologic findings in endocervical curettage or surgery for patients with a type 3 transformation zone (TZ3)

Total Pap smears (*n* = 355)	Bethesda system	Benign (*n* = 218)	CIN 1/ LSIL (*n* = 68)	CIN 2/ HSIL (*n* = 17)	CIN 3/ AIS/ HSIL (*n* = 46)	Carcinoma (*n* = 6)
I (*n* = 71)	NILM	55 (77%)	11 (15%)	2 (3%)	3 (4%)	0
II-a (*n* = 47)	NILM	43 (91%)	4 (9%)	0	0	0
II-g (*n* = 2)	AGC endocervical NOS	1 (50%)	1 (50%)	0	0	0
II-p (*n* = 92)	ASC-US	67 (73%)	17 (18%)	5 (5%)	2 (2%)	1 (1%)
IIID1 (*n* = 70)	LSIL	38 (54%)	26 (37%)	3 (4%)	3 (4%)	0
IIID2 (*n = *26)	HSIL	4 (15%)	4 (15%)	5 (19%)	12 (46%)	1 (4%)
III-g (*n* = 2)	AGC endocervical, favoring neoplastic	2 (100%)	0	0	0	0
III-p (*n* = 24)	ASC-H	4 (17%)	5 (21%)	1 (4%)	12 (50%)	2 (8%)
IVa-p (*n* = 15)	HSIL	3 (20%)	0	1 (7%)	11 (73%)	0
IVa-g (*n* = 3)	AIS	1 (33%)	0	0	1 (33%)	1 (33%)
IVb-p (*n* = 2)	HSIL with features suspicious for invasion	0	0	0	2 (100%)	0
IVb-g (*n* = 1)	AIS with features suspicious for invasion	0	0	0	0	1 (100%)

*AGC* atypical glandular cells, *AIS* adenocarcinoma in situ, *ASC-H* atypical squamous cells high-grade squamous intraepithelial lesion cannot be excluded, *ASC-US* atypical squamous cells of undetermined significance, *CIN* cervical intraepithelial neoplasia, *HSIL* high-grade squamous intraepithelial lesion, *LSIL* low-grade squamous intraepithelial lesion, *NILM* negative for intraepithelial lesion or malignancy, *NOS* not otherwise specified

Colposcopic findings were analyzed in accordance with the 2011 IFCPC colposcopic terminology for the cervix [14]. Colposcopic findings were “normal” in 354 cases, “minor” in 13 cases, “major” in 43 cases, “miscellaneous” in 11 cases, “unspecific” in two cases, and “suspicious for cancer” in six cases. Data for the correlation of colposcopic and histology findings are presented in Table 4. The histology findings were “benign” in 269 cases, “CIN 1/LSIL” in 75 cases, “CIN 2/HSIL” in 23 cases, “CIN 3/AIS/HSIL” in 54 cases, and “carcinoma” in eight cases.

**Table 4:** Correlation of colposcopic and histologic findings in accordance with the 2011 IFCPC colposcopic terminology for the cervix

Colposcopic findings (*n* = 429)	Benign (*n* = 269)	CIN 1/ LSIL (*n* = 75)	CIN 2/ HSIL (*n* = 23)	CIN 3/ AIS/ HSIL (*n* = 54)	Carcinoma(*n* = 8)
Normal (*n* = 354)	233 (66%)	61 (17%)	20 (6%)	36 (10%)	4 (1%)
Abnormal (*n* = 56)					
Grade 1 (minor) (*n* = 13)	8 (62%)	3 (23%)		2 (15%)	
Grade 2 (major) (*n* = 43)	19 (44%)	8 (17%)	2 (5%)	12 (28%)	2 (5%)
Unspecific (n = 2)	1 (50%)	1 (50%)			
Suspicious for invasion (*n* = 6)	1 (17%)			3 (50%)	2 (33%)
Miscellaneous (*n* = 11)	7 (64%)	2 (18%)	1 (9%)	1 (9%)	

*AIS* adenocarcinoma in situ, *CIN* cervical intraepithelial neoplasia, *HSIL* high-grade squamous intraepithelial lesion, *IFCPC* International Federation for Cervical Pathology and Colposcopy, *LSIL* low-grade squamous intraepithelial lesion

Sixty-one patients were younger than 35 years, while 368 women were aged 35 or older. The Pap and histology results in women aged < 35 years and in those aged 35 years or older are shown in in the supplementary materials (Tables S6 and S7).

The influence of cytology on the histologic results was assessed using two GEE-estimated logistic regression models (Table 5). When hrHPV status was not taken into account, LSIL did not show significant differences in comparison with benign results, whereas cytology suggesting HSIL + (as compared to LSIL–) showed a highly elevated risk for detection of HSIL + in histology (OR 42.945; 95% CI, 20.034 to 92.060; *P* < 0.001). When hrHPV test results were taken into account, cytology suggesting HSIL + still showed an elevated risk for detection of HSIL + in histology (OR 25.79; 95% CI, 11.943 to 55.69; *P* < 0.001). The results also showed a high correlation of hrHPV positivity with HSIL + results in histology in comparison with hrHPV negativity (OR 4.55; 95% CI, 1.913 to 10.831; *P* < 0.001).

**Table 5:** Influence of cytology on histology assessed with two GEE-estimated logistic regression models. Model 1 shows correlations without taking hrHPV status into account; model 2 shows correlations between cytology and histology under the influence of hrHPV

	OR	95% CI	*P* value
Model 1			
Pap (reference: benign)			
LSIL	1.746	0.735; 4.147	0.207
HSIL +	42.945	20.034; 92.06	< 0.001
Unspecific	19.917	8.077; 49.114	< 0.001
Model 2			
Pap (reference: benign			
LSIL	1.32	0.547; 3.18	0.537
HSIL +	25.79	11.943; 55.69	< 0.001
Unspecific	16.03	6.042; 42.511	< 0.001
hrHPV (reference: negative)			
hrHPV positive	4.55	1.913; 10.813	0.001

*CI* confidence intervals, *GEE* generalized estimating equation, *hrHPV* high-risk human papillomavirus, *HSIL* high-grade squamous intraepithelial lesion, *LSIL* low-grade squamous intraepithelial lesion, *OR* odds ratio

## Discussion

This retrospective single-center study includes 429 colposcopies with histology samples obtained using ECC. The results of Pap smears, hrHPV testing, and histology were compared in these patients and correlations with age, TZ, and colposcopic findings were assessed.

A newly organized screening program for cervical cancer was implemented in Germany in January 2020 [8, 30–33]. Women between 20 and 34 years of age continue to have annual Pap smears, while women over the age of 34 receive a co-test comprising a Pap smear in combination with a hrHPV test every 3 years. The differences between these two different age groups were therefore analyzed.

A recent registry study by the Working Group on Cervical Pathology and Colposcopy (AGCPC) assessed the diagnostic algorithm for this new cervical cancer screening system in Germany, including the data for 4763 patients. The results showed that, even in patients referred for colposcopy due to hrHPV positivity with low-risk cytology, HSIL + lesions, including AIS, were detected unexpectedly often. In patients with Pap I and hrHPV positivity, CIN 2 + lesions were found in 13.1%, while one patient had invasive cervical carcinoma [34].

Data on routinely performed ECC is inconclusive. Studies suggest that the rate of additional diagnosis of CIN 2 + by means of ECC is between 1 and 13% [20, 21]. The benefits of ECC may be greater in older women [21]. Other studies report that changes in treatment plans on the basis of additional ECC are only made in a small group of patients [18, 22].

Taking hrHPV positivity into consideration, one study identified CIN 2 + results in ECC in otherwise normal colposcopies in 11% of patients with HPV-16 and normal cytology, suggesting that ECC should potentially be carried out routinely in patients with HPV-16, normal cytology, and normal colposcopy [35]. A retrospective study including patients who had undergone ECC for endocervical lesions that were incompletely visible on colposcopy or inaccessible to biopsy, had atypical glandular cells on the smear, or discrepancies between the colposcopic impression and cytologic abnormalities, reported high sensitivity and specificity rates as well as high positive and negative predictive values and no significant increase in the risk of diagnostic underestimation with ECC [36]. The authors concluded that ECC is a reliable tool for reducing overtreatment, without increasing the risk of disease progression.

The present study adds to the existing data. It shows that the incidence of high-risk HPV positivity is significantly increased with high-grade cytologic abnormalities. In accordance with the ASCCP guidelines, ECC is recommended for all patients who are undergoing colposcopy due to a known positive test for HPV types 16 or 18. This recommendation is consensus-based and was made on the basis of moderate evidence [19]. While the patient population in the current analysis might not be large enough to fully support this conclusion, the data indicate that performing ECC should be considered in patients with hrHPV positivity and normal cytology. According to our data, out of 354 patients who presented with normal colposcopic findings, 56 showed histologic HSIL and 4 had invasive carcinoma diagnosed via ECC.

In the current study, the majority of patients presented with TZ3. This is likely associated with age. 355 women presented with TZ3 and 368 women were aged 35 years and older. In the analysis of cases with TZ3, evidence of high-grade lesions and invasive carcinoma was also found in patients with normal or low-grade abnormal cytology, supporting the hypothesis that these women benefit from ECC [16]. In accordance with the ASCCP guidelines, ECC is recommended for all patients in whom the squamous-cellular junction cannot be fully visualized at colposcopy, with moderate evidence based on a nonrandomized trial [19]. The present study assessed correlations between colposcopic findings and histology in accordance with the 2011 IFCPC colposcopic terminology for the cervix [14]. CIN 2/HSIL and CIN 3/AIS/HSIL were found in 56 cases and invasive carcinoma in four cases in patients with normal colposcopic findings. No other means such as the application of estrogen or misoprostol for a potentially better evaluation of TZ3 were used in the present patients.

The present analysis included significantly higher numbers of women over the age of 35, which is partly due to the screening program in Germany and the guidelines for colposcopy. Given that older women more often present with a TZ3 than younger women, this patient population will likely benefit more from ECC. The ASCCP guidelines state that ECC is preferred for patients aged 40 and older who are undergoing colposcopy, arguing that this patient population is at higher risk for cervical precancerous lesions or invasive cancer, as they have not been affected by the risk reduction resulting from HPV vaccination [19].

In view of the performance, availability, and cost–benefit effects of ECC, it is an inexpensive, quick, and uncomplicated diagnostic method. However, the examination is associated with inconvenience for the patient involving pain, bleeding, etc. A detailed explanation of the indication for and importance of ECC, the procedure, and potential complications of it should be provided in every case.

### Strengths and limitations

This study has the limitations involved in the inherent bias of any retrospective study. All cases of colposcopy in which ECC was not performed were excluded. This eliminated potential false-negative colposcopies. The cytologic and histologic findings were analyzed in the same department, in some cases by the same examiner. The cytologists and pathologists were aware of the colposcopic findings and thus had a potential bias influencing the cytology and histology results. Particularly in the case of higher-grade cytology, only small numbers of cases are included in the present analysis, thus allowing only limited conclusions to be drawn.

The present study assessed the more severe histology, if ECC and surgery were performed. This could lead to a potential bias over- or underestimating the predictive value of ECC. However, this bias appears to be of limited relevance. Overall, 78 patients received additional surgery. Of these patients, 22 patients had normal histologic results via ECC but abnormal results in the surgical specimen. 14 patients had CIN III/HSIL and one patient had a histology of AIS. Thus, a total of 15 patients of 413 overall patients had a relevant difference between results of ECC and surgery, indicating a potential limitation of ECC value.

In the present study, some groups in which ECC is indicated in accordance with the ASCCP recommendations were not specifically considered. This includes patients with a history of HSIL during follow-up visits. Here, ECC is recommended for all patients previously treated for CIN 2 + , regardless of the indication for colposcopy. Another recommended use of ECC is in patients who present with CIN 2/HSIL and are considered eligible for an observational regimen. In these patients, the ASCCP recommends standard ECC [19]. The potential benefits of ECC in these patient populations were not assessed in the present study and should be taken into consideration in future research.

## Conclusion

Overall, the existing data on the value of ECC in colposcopy are inconclusive and there is a lack of randomized and controlled studies. However, ECC should be considered for certain clinical patterns such as inadequate or unrepresentative colposcopy, TZ3, or postmenopausal status in the presence of abnormal cytology and/or hrHPV positivity, abnormal glandular cells, suspected invasion, or atypical glandular patterns.

The present analysis suggests that ECC is a valuable tool in the diagnosis of cervical intraepithelial neoplasia, especially for patients who present with normal colposcopy findings in the cervix and vagina but have either recurrent abnormal cytologic findings or high-grade abnormal cytology indicating CIN 2 + . The data indicate that performing ECC should be considered in patients with hrHPV positivity and normal cytology. In the analysis of cases with TZ3, evidence of high-grade lesions and invasive carcinoma was also found in patients with normal or low-grade abnormal cytology, supporting the hypothesis that these women benefit from ECC.

## Supplementary Information

Below is the link to the electronic supplementary material.

Supplementary file1 (DOCX 344 KB)

## Data Availability

The data supporting the findings of this study are available from the corresponding author upon reasonable request.
