# Supplementary material for: Evaluation of endocervical curettage (ECC) in colposcopy for detecting cervical intraepithelial lesions
Source: Arch Gynecol Obstet. 2024 Oct 29;310(6):3037–45. doi: 10.1007/s00404-024-07721-w (PMC11910394; doi:10.1007/s00404-024-07721-w)

**Evaluation of endocervical curettage (ECC) in colposcopy for detecting cervical intraepithelial lesions**

**Supplementary Material**

**Table S1** Subdivisions of Papanicolaou smear (Pap) groups for statistical analysis

| Subdivision | Munich III | Bethesda |
| --- | --- | --- |
| Benign | I | NILM |
|  | II-a | NILM |
|  | II-g | AGC; NOS |
|  | II-p | ASC-US |
| LSIL | IIID1 | LSIL |
| HSIL+ | IIID2 | HSIL |
|  | IVa-p | HSIL |
|  | IVa-g | AIS |
|  | IVb-p | HSIL with features suspicious for invasion |
|  | IVb-g | AIS with features suspicious for invasion |
|  | V-e | Endometrial adenocarcinoma |
|  | V-g | Endocervical adenocarcinoma |
|  | V-p | Squamous cell carcinoma |
|  | V-x | Other malignant neoplasms |
| Unspecific | III-e | AGC endometrial |
|  | III-g | AGC endocervical favoring neoplasia |
|  | III-p | ASC-H |
|  | III-x | AGC favoring neoplasia |

AGC, atypical glandular cells; AIS, adenocarcinoma in situ; ASC-H, atypical glandular cells of undetermined significance, HSIL cannot be excluded; ASC-US, atypical squamous cells of undetermined significance; HSIL, high-grade squamous intraepithelial lesion; LSIL, how-grade squamous intraepithelial lesion; NILM, negative for intraepithelial lesions or malignancy; NOS, not otherwise specified.

**Table****S2** Papanicolaou smear (Pap) and human papillomavirus (HPV) in combination with histology in endocervical curettage or surgery

| Total Pap smears (n = 429, 413 patients) | Bethesda system | hrHPV-positive (n = 249) | hrHPV-negative (n = 180) |  |  | Benign (n = 269) | CIN 1/ LSIL (n = 75) | CIN 2/ HSIL (n = 23) | CIN 3/ AIS/ HSIL (n = 54) | Carcinoma (n = 8) |
| --- | --- | --- | --- | --- | --- | --- | --- | --- | --- | --- |
| I (n = 84) | NILM | 36 (43%) |  |  |  | 27 (75%) | 5 (14%) | 2 (6%) | 2 (6%) |  |
| I (n = 84) | NILM |  | 48 (57%) |  |  | 41 (85%) | 6 (13%) |  | 1 (2%) |  |
| II-a (n = 59) | NILM | 15 (25%) |  |  |  | 12 (80%) | 3 (20%) |  |  |  |
| II-a (n = 59) | NILM |  | 44 (75%) |  |  | 40 (91%) | 3 (7%) | 1 (2%) |  |  |
| II-g (n = 4) | AGC endocervical NOS | 2 (50%) |  |  |  | 1 (50%) | 1 (50%) |  |  |  |
| II-g (n = 4) | AGC endocervical NOS |  | 2 (50%) |  |  | 2 (100%) |  |  |  |  |
| II-p (n = 108) | ASC-US | 60 (56%) |  |  |  | 43 (72%) | 9 (15%) | 5 (8%) | 2 (3%) | 1 (2 (%) |
| II-p (n = 108) | ASC-US |  | 48 (44%) |  |  | 37 (77%) | 9 (19%) | 2 (4%) |  |  |
| IIID1 (n = 86) | LSIL | 58 (67%) |  |  |  | 32 (55%) | 18 (31%) | 4 (7%) | 3 (5%) |  |
| IIID1 (n = 86) | LSIL |  | 28 (33%) |  |  | 16 (57%) | 11 (39%) | 1 (4%) | 1 (4%) |  |
| IIID2 (n = 33) | HSIL | 32 (97%) |  |  |  | 6 (19%) | 3 (9%) | 6 (19%) | 16 (50%) | 1 (3%) |
| IIID2 (n = 33) | HSIL |  | 1 (3%) |  |  |  | 1 (100%) |  |  |  |
| III-g (n = 2) | AGC endocervical, favoring neoplastic | 1 (50%) |  |  |  | 1 (100%) |  |  |  |  |
| III-g (n = 2) | AGC endocervical, favoring neoplastic |  | 1 (50%) |  |  | 1 (100%) |  |  |  |  |
| III-p (n = 25) | ASC-H | 19 (76%) |  |  |  | 2 (11%) | 5 (26%) |  | 10 (53%) | 2 (10%) |
| III-p (n = 25) | ASC-H |  | 6 (24%) |  |  | 2 (33%) | 1 (17%) | 1 (17%) | 2 (33%) |  |
| IVa-p (n = 19) | HSIL | 18 (95%) |  |  |  | 4 (22%) |  | 1 (11%) | 13 (72%) |  |
| IVa-p (n = 19) | HSIL |  | 1 (5%) |  |  | 1 (100%) |  |  |  |  |
| IVa-g (n = 3) | AIS | 2 (67%) |  |  |  |  |  |  | 1 (50%) | 1 (50%) |
| IVa-g (n = 3) | AIS |  | 1 (33%) |  |  | 1 (100%) |  |  |  |  |
| IVb-p (n = 2) | HSIL with features suspicious for invasion | 2 (100%) |  |  |  |  |  |  | 2 (100%) |  |
| IVb-p (n = 2) | HSIL with features suspicious for invasion |  | 0 (0%) |  |  |  |  |  |  |  |
| IVb-g (n = 1) | AIS with features suspicious for invasion | 1 (100%) |  |  |  |  |  |  |  | 1 (100%) |
| IVb-g (n = 1) | AIS with features suspicious for invasion |  | 0 (0%) |  |  |  |  |  |  |  |
| V-p (n = 2) | Squamous cell carcinoma | 2 (100%) |  |  |  |  |  |  | 1 (50%) | 1 (50%) |
| V-p (n = 2) | Squamous cell carcinoma |  | 0 (0%) |  |  |  |  |  |  |  |
| V-x (n = 1) | Other malignant neoplasms | 1 (100%) |  |  |  |  |  |  |  | 1 (100%) |
| V-x (n = 1) | Other malignant neoplasms |  | 0 (0%) |  |  |  |  |  |  |  |

AGC, atypical glandular cells; AIS, adenocarcinoma in situ; ASC-H, atypical squamous cells, high-grade squamous intraepithelial lesion cannot be excluded; ASC-US, atypical squamous cells of undetermined significance; CIN, cervical intraepithelial neoplasia; hrHPV, high-risk human papillomavirus; HSIL, high-grade squamous intraepithelial lesion; LSIL, low-grade squamous intraepithelial lesion; NILM, negative for intraepithelial lesion or malignancy; NOS, not otherwise specified.

**Table****S3**Papanicolaou smear (Pap) and human papillomavirus (HPV) in combination with histological findings in endocervical curettage or surgery for patients with a type 1 transformation zone (TZ1)

| Total Pap smears (n = 25) | Bethesda system | hrHPV-positive (n = 10) | hrHPV-negative (n = 15) |  |  | Benign (n = 18) | CIN 1/ LSIL (n = 1) | CIN 2/ HSIL (n = 1) | CIN 3/ AIS/ HSIL (n = 4) | Carcinoma (n = 1) |
| --- | --- | --- | --- | --- | --- | --- | --- | --- | --- | --- |
| I (n = 3) | NILM | 1 (33 %) |  |  |  | 1 (100 %) |  |  |  |  |
| I (n = 3) | NILM |  | 2 (67 %) |  |  | 2 (100 %) |  |  |  |  |
| II-a (n = 6) | NILM | 1 (17 %) |  |  |  | 1 (100 %) |  |  |  |  |
| II-a (n = 6) | NILM |  | 5 (83 %) |  |  | 5 (100 %) |  |  |  |  |
| II-g (n = 1) | AGC endocervical NOS | 1 (100 %) |  |  |  | 1 (100 %) |  |  |  |  |
| II-g (n = 1) | AGC endocervical NOS |  | 0 (0 %) |  |  |  |  |  |  |  |
| II-p (n = 4) | ASC-US | 2 (50 %) |  |  |  | 1 (50 %) |  | 1 (50 %) |  |  |
| II-p (n = 4) | ASC-US |  | 2 (50 %) |  |  | 2 (100 %) |  |  |  |  |
| IIID1 (n = 3) | LSIL | 1 (33 %) |  |  |  | 1 (100 %) |  |  |  |  |
| IIID1 (n = 3) | LSIL |  | 2 (67 %) |  |  | 2 (100 %) |  |  |  |  |
| IIID2 (n = 4) | HSIL | 1 (25 %) |  |  |  |  |  |  | 1 (100 %) |  |
| IIID2 (n = 4) | HSIL |  | 3 (75 %) |  |  | 1 (33 %) |  |  | 2 (67 %) |  |
| III-p (n = 1) | ASC-H | 1 (100 %) |  |  |  |  | 1 (100 %) |  |  |  |
| III-p (n = 1) | ASC-H |  | 0 (0 %) |  |  |  |  |  |  |  |
| IVa-p (n = 1) | HSIL | 0 (0 %) |  |  |  |  |  |  |  |  |
| IVa-p (n = 1) | HSIL |  | 1 (100 %) |  |  | 1 (100 %) |  |  |  |  |
| V-p (n = 2) | Squamous cell carcinoma | 2 (100 %) |  |  |  |  |  |  | 1 (50 %) | 1 (50 %) |
| V-p (n = 2) | Squamous cell carcinoma |  | 0 (0 %) |  |  |  |  |  |  |  |

AGC, atypical glandular cells; AIS, adenocarcinoma in situ; ASC-H, atypical squamous cells, high-grade squamous intraepithelial lesion cannot be excluded; ASC-US, atypical squamous cells of undetermined significance; CIN, cervical intraepithelial neoplasia; hrHPV, high-risk human papillomavirus; HSIL, high-grade squamous intraepithelial lesion; LSIL, low-grade squamous intraepithelial lesion; NILM, negative for intraepithelial lesion or malignancy; NOS, not otherwise specified.

**Table S4**Papanicolaou smear (Pap) and human papillomavirus (HPV) in combination with histological findings in endocervical curettage or surgery for patients with a type 2 transformation zone (TZ2)

| Total Pap smears (n = 49) | Bethesda system | hrHPV-positive (n = 32) | hrHPV-negative (n = 17) |  |  | Benign (n = 33) | CIN 1/ LSIL (n = 6) | CIN 2/ HSIL (n = 5) | CIN 3/ AIS/ HSIL (n = 4) | Carcinoma (n = 1) |
| --- | --- | --- | --- | --- | --- | --- | --- | --- | --- | --- |
| I (n = 10) | NILM | 4 (40 %) |  |  |  | 4 (100 %) |  |  |  |  |
| I (n = 10) | NILM |  | 6 (60 %) |  |  | 6 (100 %) |  |  |  |  |
| II-a (n = 6) | NILM | 2 (33 %) |  |  |  | 1 (50 %) | 1 (50 %) |  |  |  |
| II-a (n = 6) | NILM |  | 4 (67 %) |  |  | 2 (50 %) | 1 (25 %) | 1 (25%) |  |  |
| II-g (n = 1) | AGC endocervical NOS | 0 (0 %) |  |  |  |  |  |  |  |  |
| II-g (n = 1) | AGC endocervical NOS |  | 1 (100 %) |  |  | 1 (100 %) |  |  |  |  |
| II-p (n = 12) | ASC-US | 8 (67 %) |  |  |  | 7 (88 %) |  | 1 (12 %) |  |  |
| II-p (n = 12) | ASC-US |  | 4 (33 %) |  |  | 3 (75 %) | 1 (25 %) |  |  |  |
| IIID1 (n = 13) | LSIL | 11 (85 %) |  |  |  | 5 (45 %) | 3 (27 %) | 2 (18 %) | 1 (9 %) |  |
| IIID1 (n = 13) | LSIL |  | 2 (15 %) |  |  | 2 (100 %) |  |  |  |  |
| IIID2 (n = 3) | HSIL | 3 (100 %) |  |  |  | 1 (33 %) |  | 1 (33 %) | 1 (33 %) |  |
| IIID2 (n = 3) | HSIL |  | 0 (0 %) |  |  |  |  |  |  |  |
| IVa-p (n = 3) | HSIL | 3 (100 %) |  |  |  | 1 (33 %) |  |  | 2 (67 %) |  |
| IVa-p (n = 3) | HSIL |  | 0 (0 %) |  |  |  |  |  |  |  |
| V-x (n = 1) | Other malignant neoplasms | 1 (100 %) |  |  |  |  |  |  |  | 1 (100 %) |
| V-x (n = 1) | Other malignant neoplasms |  | 0 (0 %) |  |  |  |  |  |  |  |

AGC, atypical glandular cells; AIS, adenocarcinoma in situ; ASC-H, atypical squamous cells, high-grade squamous intraepithelial lesion cannot be excluded; ASC-US, atypical squamous cells of undetermined significance; CIN, cervical intraepithelial neoplasia; hrHPV, high-risk human papillomavirus; HSIL, high-grade squamous intraepithelial lesion; LSIL, low-grade squamous intraepithelial lesion; NILM, negative for intraepithelial lesion or malignancy; NOS, not otherwise specified.

**Table S5**Papanicolaou smear (Pap) and human papillomavirus (HPV) in combination with histological findings in endocervical curettage or surgery for patients with a type 3 transformation zone (TZ3)

| Total Pap smears (n = 355) | Bethesda system | hrHPV-positive (n = 204) | hrHPV-negative (n = 151) |  | Benign (n = 218) | CIN 1/ LSIL (n = 68) | CIN 2/ HSIL (n = 17) | CIN 3/ AIS/ HSIL (n = 46) | Carcinoma (n = 6) |
| --- | --- | --- | --- | --- | --- | --- | --- | --- | --- |
| I (n = 71) | NILM | 31 (44 %) |  |  | 22 (71 %) | 5 (16 %) | 2 (6 %) | 2 (6 %) |  |
| I (n = 71) | NILM |  | 40 (56 %) |  | 33 (83 %) | 6 (15 %) |  | 1 (2 %) |  |
| II-a (n = 47) | NILM | 12 (26 %) |  |  | 10 (83 %) | 2 (17 %) |  |  |  |
| II-a (n = 47) | NILM |  | 35 (71 %) |  | 33 (94 %) | 2 (6 %) |  |  |  |
| II-g (n = 2) | AGC endocervical NOS | 1 (50 %) |  |  |  | 1 (100 %) |  |  |  |
| II-g (n = 2) | AGC endocervical NOS |  | 1 (50 % ) |  | 1 (100 %) |  |  |  |  |
| II-p (n = 92) | ASC-US | 50 (54 %) |  |  | 35 (70 %) | 9 (18 %) | 3 (6 %) | 2 (4 %) | 1 (2 %) |
| II-p (n = 92) | ASC-US |  | 42 (46 %) |  | 32 (76 %) | 8 (19 %) | 2 (5 %) |  |  |
| IIID1 (n = 70) | LSIL | 46 (66 %) |  |  | 26 (57 %) | 15 (33 %) | 2 (4 %) | 3 (7 %) |  |
| IIID1 (n = 70) | LSIL |  | 24 (34 %) |  | 12 (50 %) | 11 (46 %) | 1 (4 %) |  |  |
| IIID2 (n = 26) | HSIL | 25 (96 %) |  |  | 4 (16 %) | 3 (12 %) | 5 (20 %) | 12 (48 %) | 1 (4 %) |
| IIID2 (n = 26) | HSIL |  | 1 (4 %) |  |  | 1 (100 %) |  |  |  |
| III-g (n = 2) | AGC endocervical, favoring neoplastic | 1 (50 %) |  |  | 1 (100 %) |  |  |  |  |
| III-g (n = 2) | AGC endocervical, favoring neoplastic |  | 1 (50 %) |  | 1 (100 %) |  |  |  |  |
| III-p (n = 24) | ASC-H | 18 (75 %) |  |  | 2 (11 %) | 4 (24 %) |  | 10 (55 %) | 2 (11 %) |
| III-p (n = 24) | ASC-H |  | 6 (25 %) |  | 2 (33 %) | 1 (17 %) | 1 (17 %) | 2 (33 %) |  |
| IVa-p (n = 15) | HSIL | 15 (100 %) |  |  | 3 (20 %) |  | 1 (7 %) | 11 (73 %) |  |
| IVa-p (n = 15) | HSIL |  |  |  |  |  |  |  |  |
| IVa-g (n = 3) | AIS | 2 (67 %) |  |  |  |  |  | 1 (50 %) | 1 (50 %) |
| IVa-g (n = 3) | AIS |  | 1 (33 %) |  | 1 (100 %) |  |  |  |  |
| IVb-p (n = 2) | HSIL with features suspicious for invasion | 2 (100 %) |  |  |  |  |  | 2 (100 %) |  |
| IVb-p (n = 2) | HSIL with features suspicious for invasion |  |  |  |  |  |  |  |  |
| IVb-g (n = 1) | AIS with features suspicious for invasion | 1 (100 %) |  |  |  |  |  |  | 1 (100 %) |
| IVb-g (n = 1) | AIS with features suspicious for invasion |  |  |  |  |  |  |  |  |

AGC, atypical glandular cells; AIS, adenocarcinoma in situ; ASC-H, atypical squamous cells, high-grade squamous intraepithelial lesion cannot be excluded; ASC-US, atypical squamous cells of undetermined significance; CIN, cervical intraepithelial neoplasia; hrHPV, high-risk human papillomavirus; HSIL, high-grade squamous intraepithelial lesion; LSIL, low-grade squamous intraepithelial lesion; NILM, negative for intraepithelial lesion or malignancy; NOS, not otherwise specified.

**Table S6** Results for Papanicolaou smear (Pap) and histology in women aged < 35 years

| Pap smears (n = 61) | hrHPV-positive (n = 33) | hrHPV-negative (n = 28) |  | Benign (n = 41) | CIN I/ LSIL (n = 7) | CIN II/ HSIL (n = 4) | CIN III/ AIS/ HSIL (n = 8) | Carcinoma (n = 1) |
| --- | --- | --- | --- | --- | --- | --- | --- | --- |
| I (n = 4) | 3 (75%) |  |  | 1 (33%) | 1 (33%) |  | 1 (33%) |  |
| I (n = 4) |  | 1 (25%) |  | 1 (100%) |  |  |  |  |
| II-a (n = 20) | 3 (15%) |  |  | 2 (67%) | 1 (33%) |  |  |  |
| II-a (n = 20) |  | 17 (85%) |  | 16 (94%) | 1 (6%) |  |  |  |
| II-g (n = 2) | 1 (50%) |  |  | 1 (100%) |  |  |  |  |
| II-g (n = 2) |  | 1 (50%) |  | 1 (100%) |  |  |  |  |
| II-p (n = 12) | 7 (58%) |  |  | 5 (71%) |  |  | 1 (14%) | 1 (14%) |
| II-p (n = 12) |  | 5 (42%) |  | 5 (100%) |  |  |  |  |
| IIID1 (n = 11) | 8 (73%) |  |  | 4 (50%) | 2 (25%) | 1 (13%) | 1 (13%) |  |
| IIID1 (n = 11) |  | 3 (27%) |  | 1 (33%) | 1 (33%) | 1 (33%) |  |  |
| IIID2 (n = 8) | 8 (100%) |  |  | 2 (25%) |  | 2 (25%) | 4 (50%) |  |
| IIID2 (n = 8) |  |  |  |  |  |  |  |  |
| III-p (n = 1) | 1 (100%) |  |  |  | 1 (100%) |  |  |  |
| III-p (n = 1) |  |  |  |  |  |  |  |  |
| IVa-p (n = 2) | 1 (50%) |  |  | 1 (100%) |  |  |  |  |
| IVa-p (n = 2) |  | 1 (50%) |  | 1 (100%) |  |  |  |  |
| IVb-p (n = 1) | 1 (100%) |  |  |  |  |  | 1 (100%) |  |
| IVb-p (n = 1) |  |  |  |  |  |  |  |  |

**Table S7**Results for Papanicolaou smear (Pap) and histology in women aged > 35 years

| Pap smears (n = 368) | hrHPV-positive (n = 216) | hrHPV-negative (n = 152) |  | Benign (n = 228) | CIN I/ LSIL (n = 68) | CIN II/ HSIL (n = 18) | CIN III/ AIS/ HSIL (n = 47) | Carcinoma (n = 7) |
| --- | --- | --- | --- | --- | --- | --- | --- | --- |
| I (n = 80) | 33 (41%) |  |  | 26 (79%) | 4 (12%) | 2 (6%) | 1 (3%) |  |
| I (n = 80) |  | 47 (59%) |  | 40 (85%) | 6 (13%) |  | 1 (2%) |  |
| II-a (n = 39) | 12 (31%) |  |  | 10 (83%) | 2 (17%) |  |  |  |
| II-a (n = 39) |  | 27 (69%) |  | 24 (89%) | 2 (7%) | 1 (4%) |  |  |
| II-g (n = 2) | 1 (50%) |  |  |  | 1 (100%) |  |  |  |
| II-g (n = 2) |  | 1 (50%) |  | 1 (100%) |  |  |  |  |
| II-p (n = 96) | 53 (55%) |  |  | 38 (72%) | 9 (17%) | 4 (8%) | 2 (4%) |  |
| II-p (n = 96) |  | 43 (45%) |  | 32 (75%) | 9 (21%) | 2 (5%) |  |  |
| IIID1 (n = 75) | 50 (67%) |  |  | 28 (56%) | 16 (32%) | 3 (6%) | 3 (6%) |  |
| IIID1 (n = 75) |  | 25 (33%) |  | 15 | 10 |  |  |  |
| IIID2 (n = 25) | 24 (96%) |  |  | 4 (17%) | 3 (13%) | 4 (17%) | 12 (50%) | 1 (4%) |
| IIID2 (n = 25) |  | 1 (4%) |  |  | 1 (100%) |  |  |  |
| III-g (n = 2) | 1 (50%) |  |  | 1 (100%) |  |  |  |  |
| III-g (n = 2) |  | 1 (50%) |  | 1 (100%) |  |  |  |  |
| III-p (n = 24) | 18 (75%) |  |  | 2 (10%) | 4 (22%) |  | 10 (53%) | 2 (10%) |
| III-p (n = 24) |  | 6 (24%) |  | 2 (33%) | 1 (17%) | 1 (17%) | 2 (33%) |  |
| IVa-p (n = 17) | 17 (100%) |  |  | 3 (18%) |  | 1 (6%) | 13 (76%) |  |
| IVa-p (n = 17) |  |  |  |  |  |  |  |  |
| IVa-g (n = 3) | 2 (67%) |  |  |  |  |  | 1 (50%) | 1 (50%) |
| IVa-g (n = 3) |  | 1 (33%) |  | 1 (100%) |  |  |  |  |
| IVb-p (n = 1) | 1 (100%) |  |  |  |  |  | 1 (100%) |  |
| IVb-p (n = 1) |  |  |  |  |  |  |  |  |
| IVb-g (n = 1) | 1 (100%) |  |  |  |  |  |  | 1 (100%) |
| IVb-g (n = 1) |  |  |  |  |  |  |  |  |
| V-p (n = 2) | 2 (100%) |  |  |  |  |  | 1 (50%) | 1 (50%) |
| V-p (n = 2) |  |  |  |  |  |  |  |  |
| V-x (n = 1) | 1 (100%) |  |  |  |  |  |  | 1 (100%) |
| V-x (n = 1) |  |  |  |  |  |  |  |  |

**Figure S1. Receiver operating characteristic (ROC) analysis with areas under the ROC curve (AUC)**

**
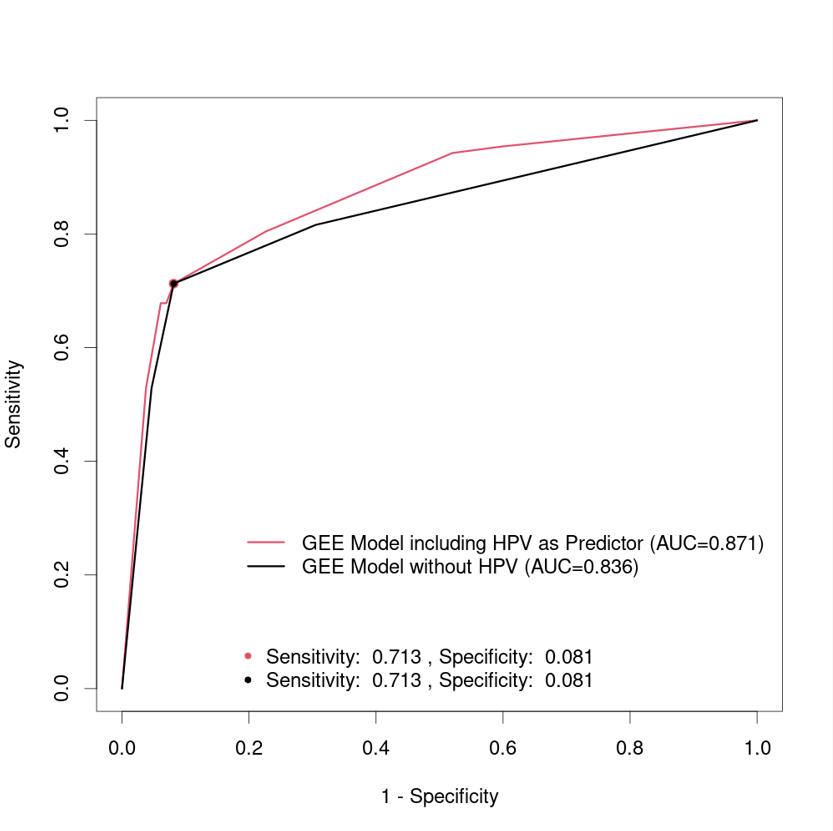
**

**Figure S2. Bootstrap Analyis with areas under the ROC curve (AUC): curves of 10000 bootstrap iterations of each model are plotted and their respective mean ROC curve together with AUC is shown**


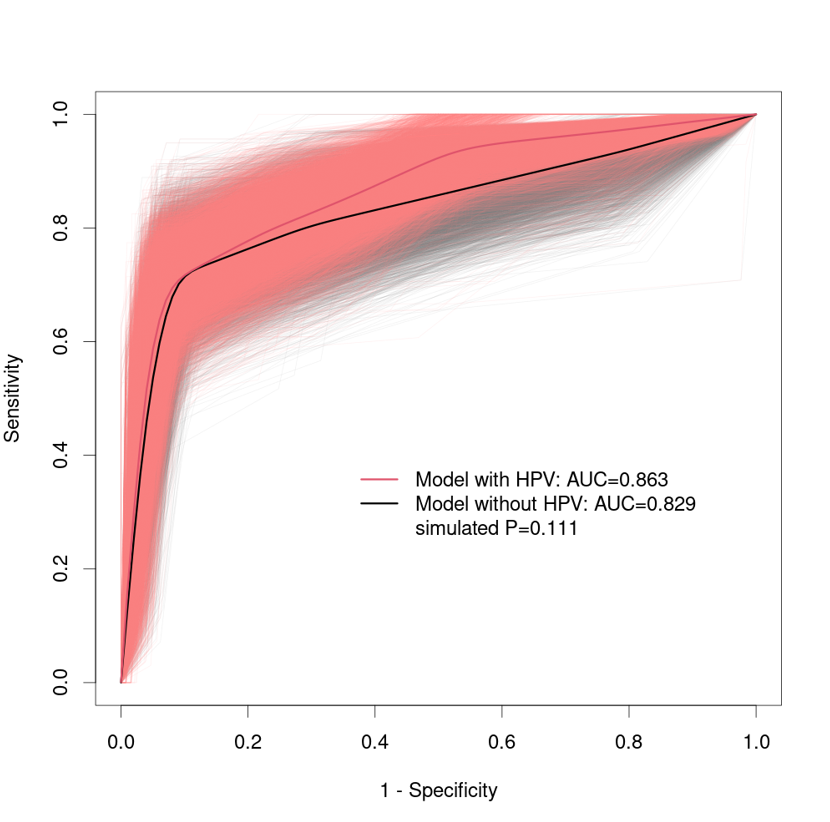

Supplement: Supplementary file 1 — Supplementary file1 (DOCX 344 KB) [file 404_2024_7721_MOESM1_ESM.docx]
